# ORAL CONCURRENT SESSION 2: Clinical Obstetrics and Quality

**DOI:** 10.1002/pmf2.12005

**Published:** 2025-01-05

Abstracts 19 – 28

THURSDAY

January 30, 2025

1:30 PM – 4:00 PM

Aurora Ballroom B

MODERATORS

Peter S. Bernstein, MD, MPH

Manisha Gandhi, MD

